# Supplementary material for: EEG-representational geometries and psychometric distortions in approximate numerical judgment
Source: PLoS Comput Biol. 2022 Dec 5;18(12):e1010747. doi: 10.1371/journal.pcbi.1010747 (PMC9754589; doi:10.1371/journal.pcbi.1010747)
Supplement: Attachment — Submitted filename: response_to_reviewers.pdf [file pcbi.1010747.s001.pdf]

PLOS Computational Biology - Revise and Resubmit (Major Revision)
-
EEG-Representational Geometries and Psychometric Distortions in Approximate Numerical Judgment
-
Stefan Appelhoff, Ralph Hertwig, Bernhard Spitzer

We thank the editors and the reviewers for the helpful and constructive suggestions. We believe that our revision accommodates all their suggestions for improvement in full, as detailed in our point-by-point reply to the reviews below.

###
Reviewer: 1

Comments to Author

Here the authors examine how sequentially presented numerical information is encoded in a judgment (i.e., the average of the stream was larger or smaller to a reference value) and in a choice context (i.e., the blue stream was larger on average than the red stream). Although behavioral analyses indicate that in the judgment context people compress numerical information (akin to a concave representation) and that in the choice context the follow the opposite pattern (akin to a convex representation), EEG encoding analyses reveal an "anti-compression" (convex representation) in both contexts.

The paper deals with an interesting research question. Furthermore, the analyses and description of the results are rigorous and clear. I have the following concerns/ suggestions:
###

We thank the reviewer for their positive evaluation of our manuscript.

###
1) The authors claim that in the judgment context representation is compressed. But Figure 1c speaks for a rather linear representation, at least at the average level. The model fits (Figure 1d) deviate significantly from linearity. It is not clear if the psychometric model misfits by large in some cases, giving rise to a significant k<1. Showing the decision weights per participant and performing some parameter recovery exercises will help. Additionally, can the authors identify critical trials in which the "compressed" and "anti-compressed" models make opposite predictions? This will help to further corroborate the claim that k<1 in judgment and k>1 in choice.
###

We are grateful for these thoughtful suggestions and comments. We agree that the model-estimated compression in the single-stream task (k=0.81) was not exceedingly strong, and we made adjustments to the wording in the revised manuscript as to not suggest otherwise. However,

as we now outline more clearly in the revised manuscript and in our reply below, the evidence for compression in the behavioral data was nevertheless reliable and robust.

Firstly, we wish to address the reviewer's comment that the weighting curve in the single stream curve (Fig. 1c *left*) would be descriptively linear. In response, we wish to highlight that the weighting curve does show the key characteristics of compression, which is a relatively more shallow local slope near extreme values (i.e., 1-2 and 8-9) than at intermediate values (i.e., 4-6). Indeed, when we compute the two-point slopes at the extremes (1-2 and 8-9) and compare them against those for mid-range value (4-5 and 5-6), the difference is highly significant (mean difference M=-0.033, SE=0.009; t(29)=-3.85, p<0.001, d=0.7; t-test against 0, two-tailed), which indicates robust compression. For comparison, in the dual stream task, the opposite is observed (a *steeper* local slope near extremes compared to mid-range values; mean difference M=0.013), indicating anti-compression. Thus, the key aspects of compression (in the single-stream task) and anti-compression (in the dual-stream task) could even be observed in model-free inspection of the data.

Second, related to the previous point, we have no reason to assume that the finding of k<1 in the single-stream task would have been due to a misfit of the data. The fitted model predictions (black dots in Fig. 1c *left*) align well with the empirical curve (blue lines) and capture its key characteristics (see previous point). This is also corroborated by goodness-of-fit statistics (AIC, which we now added to the revised manuscript, page 7 line 151). In fact, in terms of AIC, the model fit in the single-stream task (AIC=254.0) was even better than that in the dual-stream task (AIC=286.1; t(29)=-4.59, p<0.001, d=0.8; paired t-test, two-tailed). We now also added to the manuscript model comparisons (based on AIC) showing that a linear model (k=1) fitted the data significantly worse than our non-linear models (where k is a free parameter), both in the single stream (p=0.048) and in the dual stream task (p<0.001; see page 7 line 156 in the revised manuscript). It is implicit in these model comparisons that different values of k (i.e., compression or anti-compression) predict different choice behaviors in the same trials, which encompasses the reviewer's comment about critical or diagnostic trials. We also wish to highlight that we used the exact same stimulus sequences in the two tasks ('yoking', see page 16 line 466). Following the reviewer's suggestion, we also attach below plots of the individual participant fit results, which yield no evidence for major misfitting in individual participants, in neither of the two tasks. As per journal policy upon acceptance, these plots will also be published (as part of the reply letter along with the paper).

For parameter recovery simulations, we wish to refer to our recent behavioral study (Clarmann von Clarenau et al., 2022; https://doi.org/10.31234/osf.io/6yj4r), which used the same model and behavioral paradigm as the present EEG study, and where we found generally good parameter recoverability (r=0.85-0.95 for *k* in particular). We note for completeness that in the high-powered previous study (n=400), the compression observed in single-stream averaging was indeed somewhat stronger (k=0.67), but not very different from that observed in our present EEG study (k=0.81) which necessarily had a smaller participant sample (n=30 EEG participants).

In summary, we hope that our replies and revisions have convinced the reviewer that the compression (k<1) in the single-stream task, albeit not exceedingly strong, is a robust feature of the (behavioral) empirical data in our study.

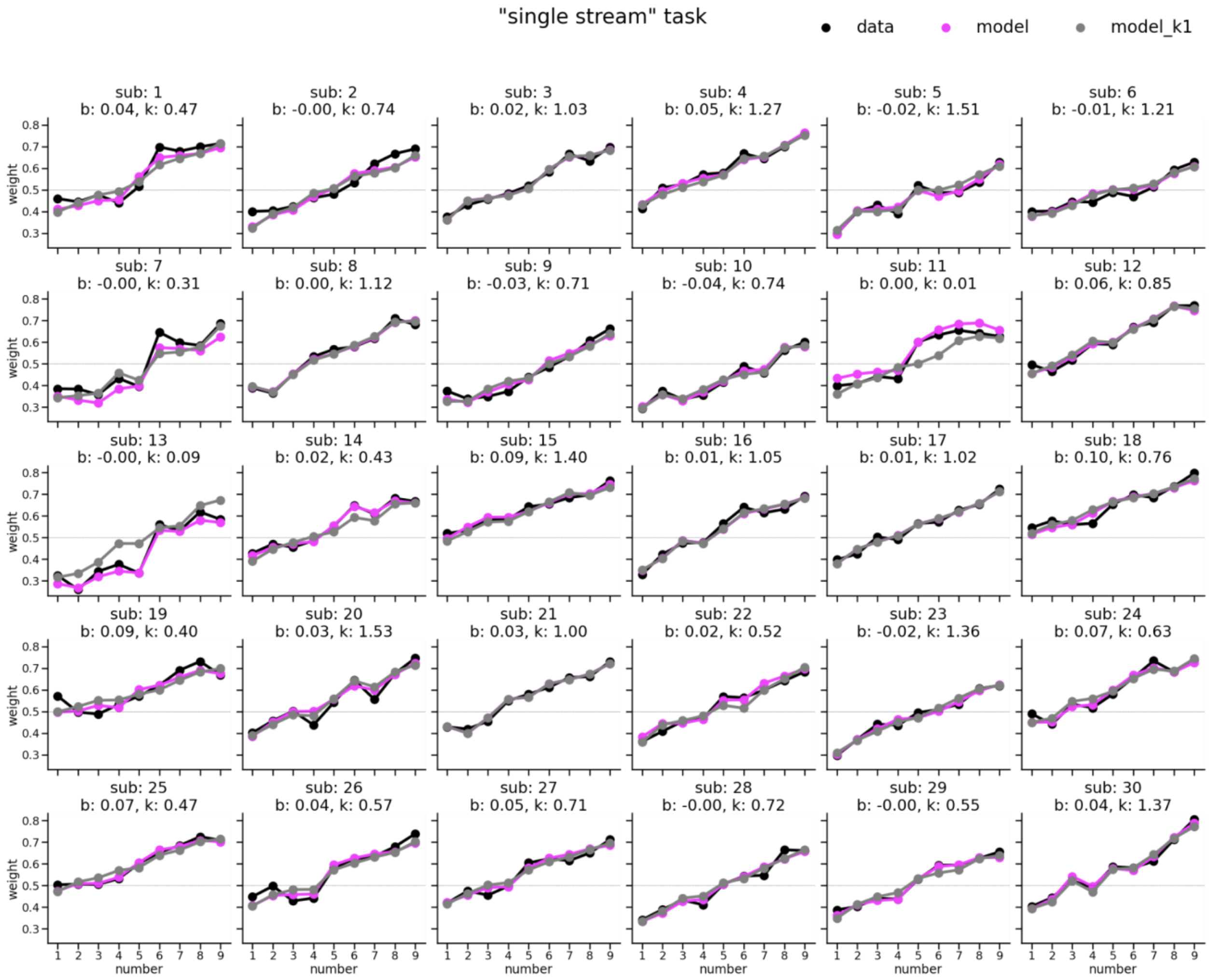

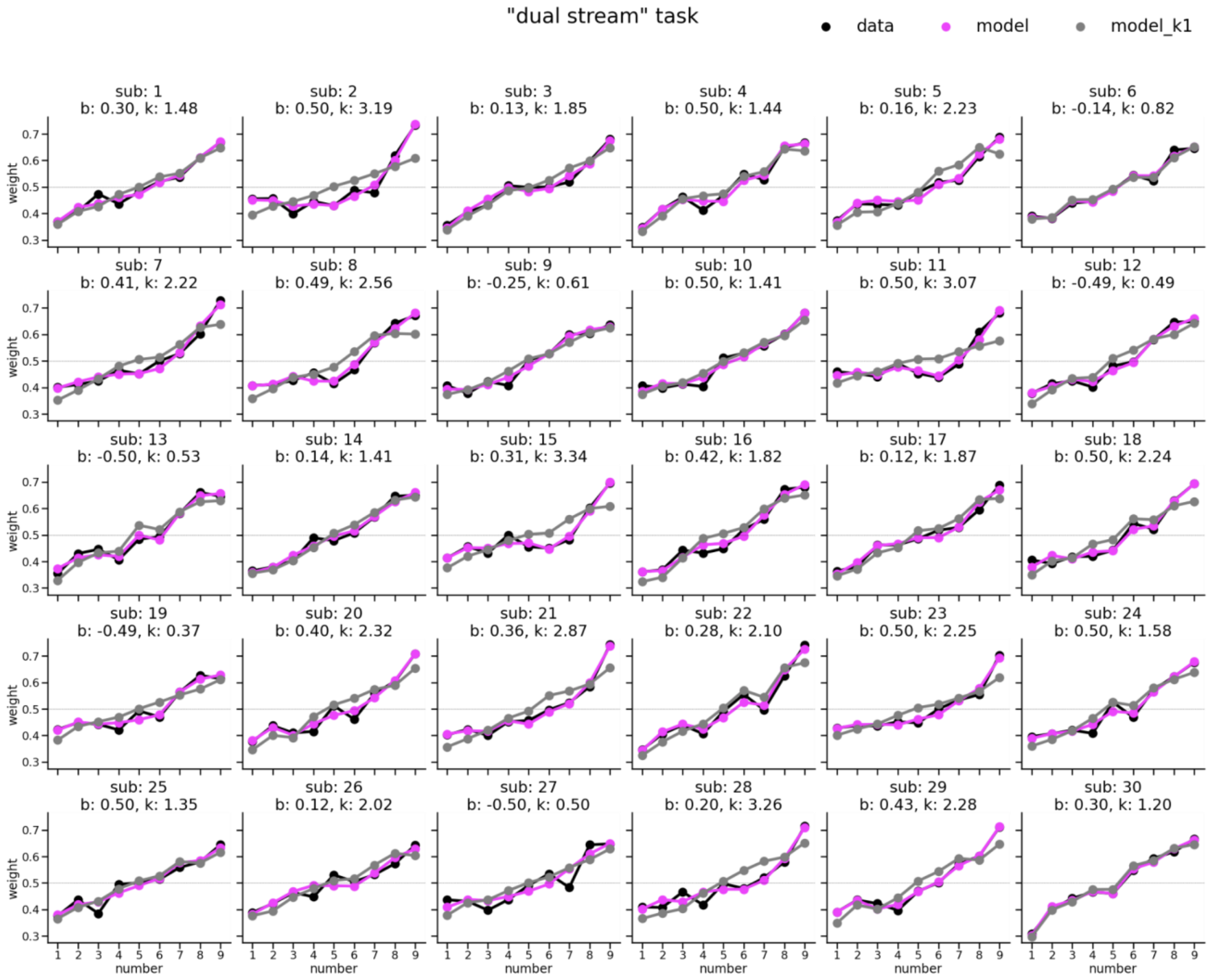

##
2) Does the order of presentation influence behavior, giving rise to primacy/ recency patterns? Additionally the authors state that "as expected, means choice accuracy was higher in the single-stream task…This suggests that comparing two streams was more difficult than averaging a single stream (of otherwise physically identical inputs)". It is not clear if the authors suggest that judgment is easier than choice. Decisions in the former are made on the basis of 10 samples whilst in the latter on the basis of 5 samples. Assuming some internal noise, the accuracy difference is not surprising. Please clarify that point and perhaps see if having the same level of internal noise (on a per sample basis) in both tasks, explains away the accuracy difference without necessitating a different value of softmax noise.
##

Here, we wish to clarify what we believe was a misunderstanding. Decisions in **both tasks** (single- and dual stream) were to be based on all 10 samples: In the dual stream task, participants were asked whether the blue samples had a higher average **than the red samples** (or vice versa). We carefully checked and revised our descriptions of the two tasks to avoid potential misunderstandings for future readers (page 5 line 109). The section now reads:

> *"In the dual-stream variant ("comparison" task), they were asked to indicate whether the red samples had a higher average value than the blue samples, or vice versa."*

We anticipate that this important clarification also addresses the reviewer's concern that the difference in accuracy (and noise) might have been a trivial consequence of fewer samples to be attended. This was not the case. Participants were always required to attend the entire stream of numbers (i.e., all 10 samples), in both tasks. Accordingly, we also have no reason to assume differences in presentation order (i.e., whether red or blue samples occurred earlier or later in the stream, which was randomized, and yoked between conditions) as an explanation for the different distortions. In the response to the reviewer's comment, we examined whether the shape (k) of the weighting functions might have differed between the first and second halves of the stream samples. We did not find this to be the case, in neither of the two tasks (both $p>0.153$, paired t-tests).

###
3) The psychometric and "neurometric" analyses do not agree. This mismatch can be an important finding, or more simply it could be the case that either of these analytical approaches is inaccurate. Importantly, the authors do not establish that the kappa & bias parameters obtained from the behavioral data correlate with the parameters obtained from the RSA analyses. Is there any association between neurometric and psychometric parameters? This comment also relates to point 1) (can the authors back up the kappa><1 conclusion using complementary behavioral analyses?).
###

We are glad that the reviewer considers our key result of a mismatch between neural and behavioral patterns an important finding. Following their suggestion, we also explored brain-behavior correlations between the psychometric and neurometric parameters. These additional explorations yielded no significant correlations, which tentatively agrees with our main conclusion of a missing link between the neural and behavioral results. We say 'tentatively' because recent work has alerted to the low validity of brain-behavior correlations with the typical sample sizes in neuroscientific studies (n<80; see e.g., Grady et al., 2020; https://doi.org/10.1002/hbm.25217). We therefore hope the reviewer approves our decision not to report the absence of correlations (although broadly consistent with our conclusions) as additional evidence for the mismatch between the neural and behavioral data patterns.

###
Reviewer: 2

Comments to Author

In this study the authors analyze human behavioral and neural responses to numbers presented in streams when subjects evaluate their average and are asked to either compare it to a reference (task 1) or to the average of another stream (task 2).

Results report a very weak evidence for two different psycho-metric distortions (compression or anti-compression) across the two tasks in the behavioral data, but a very strong evidence for the same neuro-metric distortion (anti-compression) across tasks in the neural data. The two sets of results are presented as opposite/incongruent and so they are discussed.
I think that the results are presented and discussed in a slightly distorted fashion, one that is not fully supported by the data:
###

We thank the reviewer for their assessment of our manuscript and for the valuable comments and suggestions.

###
1. Behavioral data: the authors claim that the results reported confirm "robust compression in single-stream averaging and robust anti-compression in dual stream comparison". First, the evidence for compression in the single-stream is far from robust: indeed it is based on a quite low statistic (p= 0.02), at least compared to the evidence for anti-compression in the dual task (p<0.001). Second, I could find a report of the goodness of fits of the model used to evaluate compression/anti-compressions, and from eye inspection of the plots, the single stream model might have a rather poor fit of the data. Third, what one should really compare to decide for compression vs. anti-compression are especially the extreme values. Again, from visual inspection of the data plotted, while for the dual stream it seems clear that the extreme values are overweighed, in the single stream there is extremely little evidence that the extremes are overweighed. Thus, I am far from convinced that these behavioral data are so clear-cut different across stream-types as the authors would want to report. The authors should provide more evidence for their claim for such a difference.
###

The reviewer correctly notes that with a k=0.81 and p=0.02 (in comparison to linear, k=1), the compression observed in our single-stream task was not exceedingly strong. We made several text edits throughout the revised paper as to not suggest otherwise. However, we wish to note that the effect was nevertheless statistically significant. Following the reviewer's suggestion we now also report the goodness of fits of the model in terms of AIC (page 7 line 151), which indicate that the model fit of the data in the single-stream task even *better* than that in the dual-stream task (mean AIC 254.0 vs. 286.1. We wish to thank the reviewer for this suggestion, and we hope our response alleviates concerns that the finding of compression (k<1) could have resulted from a misfit of the model.

Related to this point, we also wish to clarify the key empirical aspects of compression/anti-compression in the empirical weighting functions, in order to better guide the reviewer and future readers through visual inspection of the results in Fig. 1c (see also additions on page 5 line 118 in the revised manuscript). Compression or anti-compression can only be discerned from comparing features *within* each curve. Direct visual comparison between curves, in our experience, can potentially be misleading. The key feature of a compressed curve is that it is flattened at its extremes (i.e., 1-2 and 8-9) compared to its mid-range (i.e., 4-6). We hope that

with this explanation in mind (see also page 5 line 118 and page 7 line 166), it is clear to see even without modeling analyses that the curve in Fig. 1c *left* (single-stream) is indeed compressed. The key feature of an anti-compressed curve is the exact opposite, namely it is flattened at its midrange (4-6) compared to its extremes (1-2 and 8-9). This is what we see in Fig. 1c *right* (dual-stream). Our model, using a sign-preserving power function (page 17 line 491), captures exactly this quintessential data feature (increasingly steeper or shallower local slopes towards the extremes, compared to midrange) in a single parameter (k). For additional clarity, we improved the inset panels in Fig. 1c for better visual illustration of the curve shape differences implied by the k-estimates in our tasks. We hope the additional explanations (here and in the revised manuscript) clarify this important point, and help illustrate the quintessential difference in behavioral weighting between the two tasks (Fig. 1c)

Lastly, we wish to address the concern that the behavioral weighting functions may not have been clearly-cut different between the two tasks. We think this is a misunderstanding which may be attributable to a potential omission in our previous manuscript. We previously only reported that k was smaller than 1 (0.82, p=0.02) in the single-stream task, and larger than 1 (1.82, p<0.001) in the dual-stream task. We assumed that these <u>opposite</u> effects would be sufficient to illustrate that the k compared directly *between* the two tasks must indeed have been <u>very</u> different. In the revised manuscript, we now also included the direct statistical comparison of k between the two tasks (page 7 line 170 in the revised manuscript), which as expected is highly significant [t(29)=-6.19, p<0.001, d=1.48].

##
2. Neural data: the results are repeatedly reported in negative statements (e.g., "the neural processing of number samples in single-stream averaging was not characterized by compression), while the positive results are preceded by minimizing expressions (e.g. "but—if anything—by anti-compression"). However, the picture is not as nuanced as the authors would like to present it: for the case of the ERP analyses, for example, the results clearly and very significantly indicate that the neural processing of numbers for single AND dual streams are both and equally characterized by anti-compression.
##

The reviewer is correct that we observed clear evidence for anti-compression in Fig. 4 (ERP-analysis) also in the single-stream task. However, the picture was slightly less clear in the RSA-based neurometrics (Fig. 3; which was our analysis of interest based on previous representational "neurometric" work; see Introduction). There, we also observed anti-compression in the single-stream task, but with a grain of salt, since varying k in the single-stream task did not substantially improve the neurometric fit (page 10 line 249 and page 10 line 263). Our previous cautious wording was meant to reflect this nuance when summarizing the full pattern of results. In the revised manuscript, we made several small language edits to state the evidence for neural anti-compression more confidently, without overstating our findings, and we hope the reviewer approves of this compromise.

##

3. Discussion: the discussion is centered on the difference between the behavioral and the neural data. For the reasons reported above (point 1), I am not convinced that after all they are so different. I think that the results session and the discussion of this paper should be deeply revised.
##

We hope that our revisions, along with the detailed responses and clarifications in our replies have convinced the reviewer that the conclusions in our present manuscript are adequate, given the analysis results. In particular, we hope to have clarified that (i) there indeed was a substantial difference in behavioral psychometrics between the two tasks, which (ii) was not mirrored – and even opposed – by the neurometric results.
