# Supplementary material for: EEG-representational geometries and psychometric distortions in approximate numerical judgment
Source: PLoS Comput Biol. 2022 Dec 5;18(12):e1010747. doi: 10.1371/journal.pcbi.1010747 (PMC9754589; doi:10.1371/journal.pcbi.1010747)
Supplement: Attachment — Submitted filename: response_to_reviewers2.pdf [file pcbi.1010747.s002.pdf]

Response to the reviewer

##
Reviewer: 1

The authors have done a good job in addressing most of my comments.

##

We are pleased by the reviewer's positive evaluation of our previous revisions. We made further revisions in the manuscript, which we believe fully address the reviewer's remaining points 2) and 3). Before that, we provide a more detailed reply to his/her remaining remark about diagnostic trials (point 1), which our previous reply letter had touched upon only briefly.

##

Point 1): I am fully covered by the reply of the authors. One point that the authors have not touched upon is whether there can be some diagnostic trials (for instance low vs. high variance in the comparison task) that could provide additional corroboration for the shape of the distortion.

##

We are glad that the reviewer found his/her previous points adequately covered by our revisions. We also addressed his/her question about diagnostic trials already in our previous reply letter, albeit only succinctly. [Quoted from our previous reply letter: "It is implicit in these model comparisons that different values of k (i.e., compression or anti-compression) predict different choice behaviors in the same trials, which encompasses the reviewer's comment about critical or diagnostic trials."]

While we believe that this reply still stands, an extended answer is that since our study was designed for psychometric modeling *and* EEG-RSA (which is why we used a uniformly random distribution of sample values, see Methods; see also Spitzer et al., 2017), splitting or selection of "diagnostic" trials would need to be (i) post-hoc, (ii) very intricate, if feasible at all, and (iii) fully redundant to the modeling results we already report.

To explain, whether the choice on a trial is diagnostic of the particular distortions observed in our tasks is non-trivial, and not merely related to the variance (high or low) of samples in a trial. For example, a trial might lead to choice A under compression, and to choice B under anti-compression if it contained relatively more extreme values in favor of B, and relatively more moderate values in favor of A (or vice versa) with similar average values (in simplified terms). Moreover, which exact trials would discriminate between compressive, linear, or anti-compressive weighting will depend in intricate ways also on overall bias (as expressed in our model parameter

b). In fact, the perhaps only way we could think of attempting such post-hoc analysis would be to use our model in some way, move through its parameter space (with some arbitrary assumptions about all the parameters of non-interest), and identify trials for which the model-predicted choice would 'flip'. However, in result, such post-hoc analysis (if practicable at all), would at best be redundant with the modeling we already report.

We wish to repeat that it is at the heart of the formal model comparisons we report that different choices are predicted for the same trials, depending on the model's parameters, i.e., that our randomly created trials are "diagnostic" of these parameters (please see also previous parameter recovery analyses reported in Clarmann von Clarenau et al., 2022). Our modeling directly (and we think fully) encapsulates the critical aspect that would need to be tracked down painstakingly in any attempt of post-hoc trial selection. Our modeling does this (we think) much more elegantly, objectively quantifiably, in a way that is comparable to previous work which used the same approach (Clarmann von Clarenau et al., 2022; Li et al., 2017; Luyckx et al., 2019; Spitzer et al., 2017), and in a way that can be directly compared to analogous models of the EEG data, which was the very goal of our study.

Finally, we wish to emphasize that we have corroborated the shapes of the behavioral distortions in multiple ways, including (i) formal model comparisons, (ii) statistical analysis of parameter estimates and (iii) model-free analysis of the descriptive weighting curves (see our previous reply letter). The same pattern of behavioral distortions is also replicated in a high-powered online study using single- and dual-stream tasks in over 600 participants (Clarmann von Clarenau et al., 2022), including the finding of compression in the single-stream task (at $p<0.001$). We hope that in light of all this, the reviewer and the editors agree that our behavioral results are sufficiently corroborated by widely accepted scientific standards, both empirically and methodologically.

##

Point 2): thanks for clarifying the task details. What has not been addressed is the question of serial position effects. What I am missing is a more accurate characterisation of behaviour in the two tasks. Notably, participants find averaging easier than comparison. What could explain this result asides from assuming that the former has a lower noise level than the latter (which is a re-description of the accuracy difference)? My suggestion is to estimate the "leak" of accumulation and compare across tasks (e.g. Wyart, V., Myers, N. E., & Summerfield, C. (2015). Neural mechanisms of human perceptual choice under focused and divided attention. Journal of neuroscience, 35(8), 3485-349).

Assuming that fitting a "noise, leaky" accumulator reveals processing differences in the two tasks, then the question is how these differences map onto the parameter of the psychometric model and especially the distortion parameter. That is, if simulated data are generated by two leaky accumulators with linear input representation but different noise/ leak parameters, is the psychometric fit going to (mistakenly) reveal differences in the distortion parameters? This is largely a sense-checking step that would increase confidence in the reported behavioural results.

##

We apologize for this oversight in our previous reply. We now make clearer in the revised manuscript that whether a leakage parameter is included or not in our model in fact makes no difference for the results in distortions kappa (k), on page 18 line 536:

> "In previous studies using this model, we also included a "leakage" parameter (Wyart et al., 2015) to additionally account for recency effects in decision weighting (Clarmann von Clarenau et al., 2022; Spitzer et al., 2017). We here included no such additional parameter in the interest of parsimony, as our focus was on the overall correspondence of distortions k between EEG and behavior. Control analysis including a leakage parameter in the behavioral model fully replicated the present results for parameter k (see also Clarmann von Clarenau et al., 2022). We report for completeness that the leakage parameter was larger in the dual- than in the single-stream task ($p<0.01$), possibly related to the dual-stream task being overall harder (for detailed discussion of the processing demands in single- and dual stream tasks, see Clarmann von Clarenau et al., 2022)."

For the reviewer to verify, we show below the critical result for parameter kappa (k) from control analysis including a leakage term. The results are practically indistinguishable from those reported with the more parsimonious model in the paper, and they show at the same levels of statistical significance.

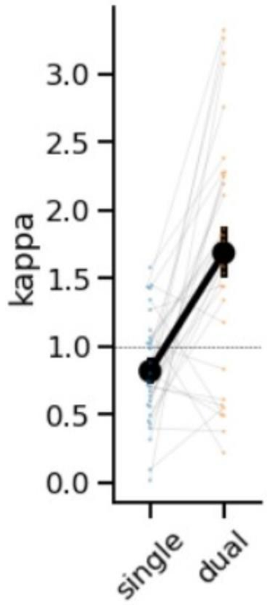

In other words, it can be ruled out that the distortions k observed in behavior could have come about artifactually somehow due to differences in leakage. In this control analysis, we actually did observe stronger leakage in the dual-stream task, which we also report (page 18 line 541).

However, this supplementary observation does not explain away anything of our distortion findings, as shown above.
Although beyond the scope of our present study (as leakage effects cannot be equivalently examined in EEG-RSA), we show for completeness that even if leakage were included in the model, it does not interact with the estimated distortions k. This can be shown in parameter recovery analysis. Below we show results (correlation matrix) from parameter recovery performed in Clarmann et al., 2022, which used the same model, tasks, and (uniform) sample distribution, but also included a leakage term (l):

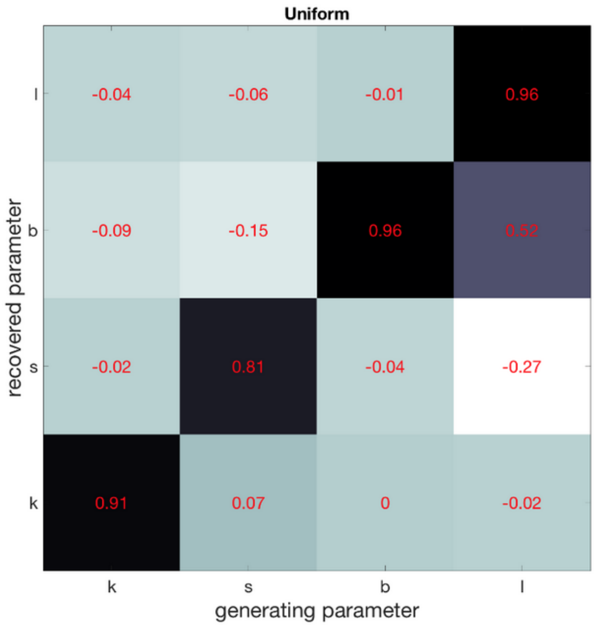

Correlations between leakage (l) and distortions kappa (k) were negligible (-0.04 and -0.02), showing that there is no crosstalk between the two in the model, while both k and l recover very well (0.91 and 0.96).

More generally, we do not consider it surprising that the dual-stream task is harder (and potentially also more prone to leakage). The dual-stream task requires integrating the interaction of two sample features (numerical value x color), whereas the single-stream task requires integrating a single sample feature only (numerical values). To provide a better intuition of this, we now also extended the text right at the beginning of our Results report (page 5 line 116):

> "Intuitively, the latter task is cognitively more demanding (see also Clarmann von Clarenau et al., 2022) as it requires evaluating the sample values according to their respective colors, whereas in the single-stream task, the colors can be ignored."

We hope this adds clarity for future readers, and we believe that the additional control analyses convincingly demonstrate that the behavioral distortions we report can not be explained away by leakage effects.

##

Point 3) I appreciate the additional analyses performed. I recommend reporting the absence of correlations (even briefly) since this strengthens the argument that the author make on the missing link between neural representations and behaviour. Given that the behavioural and neural analyses are central in this paper, exploring the relationship between the two seems an obvious step (despite the potential lack of statistical power).

##

We now briefly report these analyses also in the paper as requested by the reviewer (page 13 line 325), which reads:

> "Together, our findings indicate a lack of correspondence between the psychometric and neurometric distortions observed in our tasks. Possibly consistent with this, explorative analyses also showed no substantial correlations between our participant's individual psycho- and neurometric distortion parameters. This was especially true in the single-stream task (all r <0.26, all p>0.17, uncorrected). Anecdotally, there was an indication of a positive relation between psycho- and neurometric k only in the dual-stream task (RSA: r=0.32, p=0.08; ERP: r=0.41, p=0.03, uncorrected), which, however, was insignificant after controlling for multiple testing (all p>0.10, FDR-corrected). We exert caution in interpreting these brain-behavior correlations given a relatively small sample size (Grady et al., 2021)."

We thank the reviewer for his/her additional comments.

References

Clarmann von Clarenau, V., Pachur, T., & Spitzer, B. (2022). *Over- and Underweighting of Extreme Values in Decisions from Sequential Samples* [Preprint]. PsyArXiv. https://doi.org/10.31234/osf.io/6yj4r

Grady, C. L., Rieck, J. R., Nichol, D., Rodrigue, K. M., & Kennedy, K. M. (2021). Influence of sample size and analytic approach on stability and interpretation of brain-behavior correlations in task-related fMRI data. *Human Brain Mapping*, *42*(1), 204–219. https://doi.org/10.1002/hbm.25217

Li, V., Castañón, S. H., Solomon, J. A., Vandormael, H., & Summerfield, C. (2017). Robust averaging protects decisions from noise in neural computations. *PLOS Computational Biology*, *13*(8), e1005723. https://doi.org/10.1371/journal.pcbi.1005723

Luyckx, F., Nili, H., Spitzer, B., & Summerfield, C. (2019). Neural structure mapping in human probabilistic reward learning. *ELife*, *8*, e42816. https://doi.org/10.7554/eLife.42816

Spitzer, B., Waschke, L., & Summerfield, C. (2017). Selective overweighting of larger magnitudes during noisy numerical comparison. *Nature Human Behaviour*, *1*(8), 1–8. https://doi.org/10.1038/s41562-017-0145

Wyart, V., Myers, N. E., & Summerfield, C. (2015). Neural Mechanisms of Human Perceptual Choice Under Focused and Divided Attention. *Journal of Neuroscience*, *35*(8), 3485–3498. https://doi.org/10.1523/JNEUROSCI.3276-14.2015
